# Wild-Type Zebrafish (*Danio rerio*) Larvae as a Vertebrate Model for Diabetes and Comorbidities: A Review

**DOI:** 10.3390/ani11010054

**Published:** 2020-12-30

**Authors:** Maryna van de Venter, Jenske Didloff, Shanika Reddy, Bresler Swanepoel, Sharlene Govender, Ntokozo Shirley Dambuza, Saralene Williams, Trevor Craig Koekemoer, Luanne Venables

**Affiliations:** 1 Department of Biochemistry and Microbiology, Nelson Mandela University, PO Box 77000, Port Elizabeth 6031, South Africa; jenske.didloff@mandela.ac.za (J.D.); shanika.reddy@mandela.ac.za (S.R.); bresler.swanepoel@mandela.ac.za (B.S.); sharlene.govender@mandela.ac.za (S.G.); saralene.williams@mandela.ac.za (S.W.); trevor.koekemoer@mandela.ac.za (T.C.K.); luanne.venables@mandela.ac.za (L.V.); 2 Department of Pharmacy, Nelson Mandela University, PO Box 77000, Port Elizabeth 6031, South Africa; ntokozo.dambuza@mandela.ac.za

**Keywords:** zebrafish, wild-type, larvae, diabetes, oxidative stress, inflammation

## Abstract

**Simple Summary:**

Diabetes is predicted to become a major global pandemic in the not too distant future; subsequently, there is an urgent need to develop new drugs to treat this disease and its associated complications. Animal studies form an integral component of drug development, as they provide information concerning safety and efficacy of new drugs in an experimental setting reflecting the complexity of human physiology. Over the past decade, research has firmly established that zebrafish represent a convenient preclinical animal model. While genetic manipulation has given rise to numerous target specific models, these mutant zebrafish are unfortunately also associated with serious disadvantages. Therefore, wild-type zebrafish may be a preferred option for many researchers; however, information relating to their use in diabetes research remains scattered. This review collates existing scientific literature specifically pertaining to wild-type zebrafish and highlights the value of wild-type zebrafish larvae as a suitable animal model. Details regarding organ development, including similarities to mammalian counterparts, are discussed to indicate relevance. In addition, information covering experimental design and endpoint analysis provides an overview of the technical aspects when using wild-type zebrafish larvae as an experimental tool to mimic diabetes and its associated complications.

**Abstract:**

Zebrafish have become a popular alternative to higher animals in biomedical and pharmaceutical research. The development of stable mutant lines to model target specific aspects of many diseases, including diabetes, is well reported. However, these mutant lines are much more costly and challenging to maintain than wild-type zebrafish and are simply not an option for many research facilities. As an alternative to address the disadvantages of advanced mutant lines, wild-type larvae may represent a suitable option. In this review, we evaluate organ development in zebrafish larvae and discuss established methods that use wild-type zebrafish larvae up to seven days post fertilization to test for potential drug candidates for diabetes and its commonly associated conditions of oxidative stress and inflammation. This provides an up to date overview of the relevance of wild-type zebrafish larvae as a vertebrate antidiabetic model and confidence as an alternative tool for preclinical studies. We highlight the advantages and disadvantages of established methods and suggest recommendations for future developments to promote the use of zebrafish, specifically larvae, rather than higher animals in the early phase of antidiabetic drug discovery.

## 1. Introduction

Diabetes is a debilitating disease with a significant economic impact on health systems across the globe [1]. The International Diabetes Federation (IDF) estimated that 463 million adults were living with diabetes in 2019, with an associated USD 760 billion dollars in health care expenses [2,3].

The classical drug discovery approach starts with in vitro screening against accepted drug targets to identify lead compounds, followed by in vivo testing of only the most active samples in animal models. This approach is challenging for antidiabetic drug discovery due to the complex nature of the disease with a host of independent drug targets acting on different tissues. Defronzo (2009) defined the “ominous octet”, eight pathophysiological mechanisms underlying Type 2 diabetes that are targeted by current glucose lowering therapies [4,5]. The relatively low success rate of these treatments highlights the need for novel antidiabetic drug classes with multiple targets [6]. A strong correlation has been shown between oxidative stress, chronic inflammation, and the multitude of diabetic complications such as cardiovascular disease, diabetic retinopathy, neuropathy, nephropathy, and cancer [7,8]. Treatments that target excessive oxidative stress and chronic inflammation therefore hold great potential in reducing the incidence and severity of diabetic complications, especially in combination with anti-hyperglycaemic treatments.

Thorough in vitro antidiabetic screening requires the use of multiple enzymatic and cellular bioassays in order to fully assess the antidiabetic potential of a set of samples against all the possible drug targets. Current in vivo models for diabetes range from non-mammalian animals to non-human primates and all have their own unique advantages and disadvantages [9]. The most widely used in vivo diabetes models are rodents. This includes genetic models of Type 1 and Type 2 diabetes and diet-induced insulin resistant- and chemically induced diabetic models [10]. Although very useful, these models also have major drawbacks such as the requirement for specialized housing facilities and ethical considerations, which limit throughput capacity. The complexity of diabetes in humans and the many potential drug targets make it very difficult to select the appropriate rodent model [9]. Despite the large number of rodent models that are available, there is not a single one that truly reflects the disease as it occurs in humans [11]. Non-human primate models more closely resemble the disease in humans, but they pose greater challenges with respect to accessibility, housing facilities, and ethical considerations. The “3Rs” principle, a drive to replace, reduce, and refine animal experimentation, has forced researchers to look for alternatives to replace or reduce the number of warm-blooded animals used for testing [12].

Zebrafish (*Danio rerio*) have gained popularity as an animal model in biomedical [13], toxicological [14], and pharmacological research [15,16]. There are many advantages to using zebrafish and especially early stage larvae in biomedical research and drug discovery. According to the EU Directive for the protection of animals used for scientific purposes, these larvae are only subject to regulations for animal experimentation once they are free-living, which starts at 120 h post-fertilization (hpf) [17]. Eggs are fertilized externally, and larvae are transparent and can be stained with a wide variety of fluorescent dyes and antibodies, and they are small enough for whole-organism imaging in multiwell plates. By 96 hpf, all their major organs are fully developed. All these features contribute to them becoming more and more popular for high throughput and high content analysis [18]. The zebrafish genome has been sequenced, and it is now known that 71% of human proteins and 82% of disease-causing human proteins have an orthologue in zebrafish [16].

A number of review articles have been published on zebrafish as a model for diabetes and metabolic disease [19,20,21,22,23,24,25,26]. From this literature, it is clear that the majority of methods modelling diabetes and/or metabolic disease use adult and genetically modified fish. The zebrafish genome has been successfully manipulated using a variety of techniques such as transgenesis, mRNA injections, morpholino injections, gene disruption, and genome editing [9]. These genetically modified lines are costlier and more difficult to maintain than wild-type zebrafish. Many countries have strict regulations to protect their biodiversity, making it very tedious and sometimes almost impossible to import genetically modified models. The use of wild-type zebrafish eliminates potential complications around permits, while the use of early stage larvae rather than adults can eliminate or reduce the need for animal ethics approval. Different animal ethics regulations apply in different countries, and the requirement for ethics approval ranges from four days post fertilization (dpf) before nociception and stress is experienced [27] to five dpf when free feeding starts or even seven dpf before external food is essential for survival [28].

Considering the issues above, this review focuses on existing methods to test for antidiabetic activity using wild-type zebrafish larvae up to seven dpf. Advantages, disadvantages, and gaps that require attention will be highlighted. A list of characterized wild-type zebrafish strains that are acceptable for research purposes is available on the Zebrafish Information Network (ZFIN) website [29].

## 2. Development of Organs Associated with Diabetes in Zebrafish

**Figure 1:**
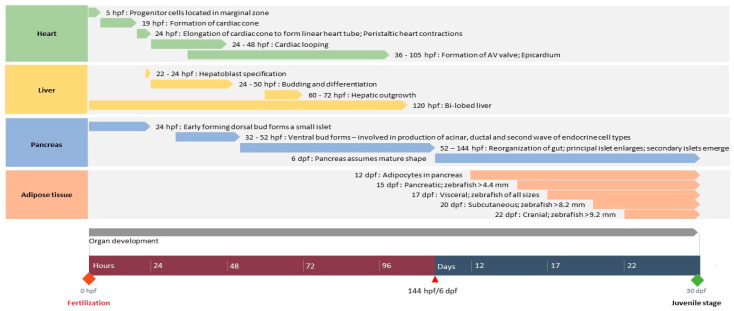
Timeline of the development of organs relevant to diabetes from fertilization of the zebrafish egg to the juvenile stage. AV: Atriovenricular.

Based on the experimental requirements and ethical restrictions on the use of zebrafish larvae, it is important to understand the development of the key organs most commonly associated or affected by diabetes mellitus and targeted by antidiabetic drugs. In this section, we will be highlighting the structural and developmental similarities and differences of zebrafish organs relevant to toxicity and diabetes at key time points during organogenesis and the early and late larval stages compared to those of humans. Figure 1 summarizes the development of key structures of the heart, liver, pancreas, and adipose tissue over the first 30 dpf.

### 2.1. Heart

#### 2.1.1. Structure

The anatomy of the zebrafish heart differs from the human heart and consists of a single atrium and ventricle that are separated by atrioventricular (AV) valves. The cellular and molecular mechanisms involved in the development are conserved between humans and zebrafish; however, the developmental time required is significantly less in zebrafish. Conserved morphological events include progenitor formation, myocardial plate and heart tube formation, cardiac looping, and formation of valves. Electrical properties of the zebrafish heart are similar to the human heart, with zebrafish embryonic heart rate of 140–180 beats per minute (bpm) closely resembling the human fetal heart rate of 130–170 bpm [30].

#### 2.1.2. Development

The zebrafish heart is composed of three cell types, namely ventricular cardiomyocytes, atrial cardiomyocytes, and endocardial cells, which originate at 5 hpf. These cells differentiate from progenitor cells in the marginal zone of the embryo [31]. The precursor cells migrate towards the embryonic midline where they reach their endpoint by 18 hpf in the anterior lateral plate mesoderm (ALPM). The cell population merges after initial contact, forming a shallow cone (19 hpf). During the migration of the myocardial cells to the midline, endocardial precursors located in the ALPM move towards the forming cone covering its interior [32]. After the formation of the cardiac cone by endocardial and myocardial precursors, the cone elongates to form a linear heart tube at 24 hpf. The heart tube exhibits peristaltic contractions by 24 hpf with sequential contraction of the ventricle and atrium occurring at 36 hpf. Between 24–48 hpf, the heart tube is looped toward the right side [33]. With the looping of the heart tube, the chambers of the heart form bulges with a bean-shaped morphology through a process known as ballooning [32,34]. This results in the displacement of the ventricle and atrium so that the ventricle is located on the right side and the atrium on the left side of the midline separated by the AV canal (AVC) [31,32]. The first notable sign of the AVC is cuboidal cells expressing cell adhesion molecules at 36 hpf and forming a single layer at 48 hpf. The endocardial cells form an endocardial cushion by the invagination of the cells that enlarges and by 105 hpf differentiates into valve leaflets that ensure unidirectional blood flow. This differs from the epithelial–mesenchymal transition in amniotes [34] in which endothelial cells reduce their markers and acquire mesenchymal markers (e.g., α-smooth muscle actin). The endothelial cells undergo delamination and invade the extracellular matrix where the cells proliferate and differentiate into valve interstitial cells (VIC). The VIC stratify to form the valve leaflet [35]. The formation of AVC by an epithelial–mesenchymal transition [31,36] or invagination [32,34,35,37] has been debated and remains controversial. The epicardium develops from the pro-epicardium composed of an extra-cardiac cell population. The spherical cells of the pro-epicardium can be recognized at 48 hpf, and by 72 hpf they expand, covering the looped heart [31,34].

#### 2.1.3. Link Between Heart and Diabetes

Diabetes causes a change in systemic and cardiac glucose metabolism that leads to functional and structural abnormalities of the heart. The combinatorial effect of hyperglycemia, insulin resistance, hypertension, dyslipidemia, and inflammation damage the endothelium, which results in microvascular damage, macrovasculopathy, and cardiovascular disease [38]. The mechanisms involved in altered function include decreased glucose transport and carbohydrate oxidation, increased utilization of free fatty acids (FFA), decreased sarcolemmal calcium transport, and a change in regulatory proteins [39].

### 2.2. Liver

#### 2.2.1. Structure

Liver development in zebrafish occurs rapidly with the formation of a functional liver within a few days, compared to weeks in humans. However, the pathways and genes involved in development are conserved [40]. The anatomy of the zebrafish liver is tubular as opposed to the lobular architecture of the human liver. The hepatocytes are arranged in tube-like cords containing a central biliary canal. Canaliculi (preductules/intrahepatic bile channels) develop as invaginations within the apical hepatocyte membrane [41]. The zebrafish liver contains most mammalian cell types, except Kupffer cells with similar functionality to humans, including the secretion of bile, lipid, and glycogen storage, insulin responsiveness, metabolism of xenobiotics, and secretion of proteins [42].

#### 2.2.2. Development

The development of the liver proceeds through three phases that include progenitor specification, budding/differentiation, and outgrowth.

Hepatic specification: Hepatic specification occurs after the anterior foregut endoderm has formed around 22–24 hpf. The liver progenitors, hepatoblasts, originate in a lateral region of the anterior foregut endoderm, where they aggregate and form a liver bud to the left [40,41]. During specification, the hepatoblasts can be detected through the expression of specific markers (hhex and prox1) [40,43].

Budding/Differentiation: Differentiation of hepatoblasts into mature hepatocytes and biliary epithelial cells occurs between 24–50 hpf. The expression of the hepatocyte markers in mammalian embryos, albumin, and alpha-fetoprotein are absent in zebrafish. Hepatocyte markers used in studies and expressed in zebrafish include ceruloplasmin (32 hpf), liver fatty acid binding protein (L-FABP), and transferrin (48 hpf) [41]. The budding phase can be divided into three stages based on morphology. During budding stage I (24 hpf), endodermal cells caudal to the pharyngeal region aggregate, forming a rod below the midline. At 28 hpf, the liver primordium is formed as an anterior section of the endodermal rod thickens. During stage II (30 hpf), the liver primordium increases in size and bends leftward, which is known as gut-looping [44,45,46]. The last stage occurs at 34 hpf with the development of a furrow between the bud and the esophagus. Posterior expansion of the furrow limits the connection between the liver and intestinal primordium. Cells joining these organs form the hepatic duct by 50 hpf with the end of stage III [44,45].

Hepatic outgrowth: Outgrowth starts between 60–72 hpf when the liver enlarges and continues to develop until it achieves the proportional size [43]. During this phase, differentiation of hepatoblasts into hepatocytes, and bile duct cells also occur. The placement of the liver also changes as it extends across the midline ventral to the oesophagus. By five days post fertilization (dpf), the liver is bilobed with a larger left lobe and smaller right lobe [45].

#### 2.2.3. Link between Liver and Diabetes

Complications of diabetes can include liver damage due to the altered metabolism of lipids, proteins, and carbohydrates due to hyperglycemia. This causes liver damage that can lead to non-alcoholic fatty liver disease, steatohepatitis, cirrhosis, and hepatocellular carcinomas [47]. Several liver abnormalities such as abnormal glycogen storage and increased hepatic enzymes are also associated with diabetes [48]. The liver damage is a combination of oxidative stress and inflammatory responses that damages hepatocytes [47].

### 2.3. Pancreas

#### 2.3.1. Structure

The zebrafish pancreas is an elongated organ that can be asymmetrically located on the right side of the body adjacent to the right side of the duodenum. It is approximately 1 mm in diameter and has two distinct colocalized compartments [49]. The exocrine compartment contains acinar cells, which produce digestive enzymes and the ducts through which they flow. The endocrine compartment contains the islets of Langerhans, which comprise insulin-secreting beta cells, antagonistically acting glucagon-secreting alpha cells, somatostatin-secreting delta cells, and a small number of ghrelin-secreting epsilon cells [19]. The major differences between zebrafish and human endocrine islets are found in the spatial arrangement where the core of the zebrafish islets is made up of both beta (~50%) and delta cells (20–25%), and the alpha cells (20–25%) are found in the periphery [49,50]. Human endocrine islets have a characteristic “mantle-core” organization of which the core consists of mainly beta cells (70–80%) and the mantle or periphery consists of non-beta cells (5% delta cells and 15–20% alpha and/or polypeptide-secreting cells) [50]. Another distinct difference is the lack of pancreatic polypeptide-secreting cells in zebrafish islets [51].

#### 2.3.2. Development

The pancreas of both mammals and zebrafish are formed from dorsal and ventral pancreatic buds [51]. However, in zebrafish, significant levels of endocrine hormones are expressed prior to the formation of the buds [49]. By 24 hpf, the early forming dorsal pancreatic bud of zebrafish forms a small islet. The ventral bud forms by 32 hpf and is involved in the production of acinar, ductal, and the second wave of endocrine cell types. Due to morphological reorganization of the gut at 52 hpf, the dorsal and ventral buds are brought together, leading to the enlargement of the principal islet [52]. As larval development continues, additional secondary islet cells and insulin-positive cells emerge along the length of the intrapancreatic duct [19], and the pancreas assumes its mature shape by 6 dpf [49]. Development of the zebrafish pancreas occurs in the same order as the human pancreas. At first, insulin-secreting principal islets form, and this is followed by the formation of glucagon-secreting cells. In mice, this pattern is reversed and therefore islet development in zebrafish more closely resembles human islet development [51].

#### 2.3.3. Link between Pancreas and Diabetes

The vertebrate pancreas plays a role in the digestion of food through exocrine contributions and in metabolic homeostasis through endocrine control. It is widely accepted that the dysfunction of endocrine beta cells often results in diabetes mellitus. Islet hormones in zebrafish, like in mammals, are required for glucose homeostasis [53]. It has been shown that insulin stimulates the uptake of glucose in zebrafish by skeletal muscle and adipose tissue as well as activates glycolysis and glycogenesis in the liver. Likewise, glucagon has been shown to stimulate the production of glucose through glycogenolysis in the liver and gluconeogenesis from lactate and amino acids, as well as from glycerol through lipolysis [49,53].

### 2.4. Adipose Tissue

#### 2.4.1. Structure

Endotherms, such as mammals, have two distinct types of adipose tissue, namely brown adipose tissue (BAT) and white adipose tissue (WAT). BAT is made up of multiple microvesicular lipid droplets as well as copious amounts of mitochondria and functions to generate heat using lipids. WAT can be described as a large unilocular lipid droplet made by the fusion of smaller lipid droplets which is stored as an energy reserve [54]. Zebrafish are poikilothermic vertebrates, as their body temperature is known to vary with ambient temperature [55]. Similarly to mammalian adipocytes, differentiated zebrafish adipocytes express acrp30, cfd, and fbp11a marker genes, responsible for the production of adipokines such as adipsin and adiponectin [56]. Although the functional role of these adipokines is still unknown, it is considered that zebrafish adipocytes are morphologically identical to mammalian adipocytes [57]. All adult zebrafish are known to contain WAT in the pancreatic, subcutaneous, visceral, oesophageal, mandibular, cranial, and tail-fin depots [56]. Zebrafish are considered to not possess BAT, even though they have been shown to have uncoupling protein 1 (UCP1) homologs associated with BAT in other species [55].

#### 2.4.2. Development

WAT is not found in zebrafish embryos, but adipocytes have been identified in the pancreas of young larvae at 12 dpf. The presence of WAT formation seems to firstly be dependent on size and then the age of the zebrafish, where temporal cues signal WAT development, regardless of size [56]. At 15 dpf, pancreatic WAT is found in zebrafish larger than 4.4 mm and at 17 dpf all fish have pancreatic and visceral WAT, regardless of size. Subcutaneous WAT appears at 20 dpf in fish larger than 8.2 mm, whereas cranial WAT appears at 22 dpf in fish larger than 9.4 mm. At 30 dpf, all zebrafish that exceed a standard length of 10.6 mm contain the adult repertoire of WAT depots [56]. At three months old, adult zebrafish have been shown to have WAT in four distinct locations, including pancreatic, visceral, subcutaneous, and cranial. Pancreatic adipocytes are intertwined with acinar cells, visceral adipocytes line the peritoneal cavity and are posterior to the swim bladder, cranial adipocytes are found in clusters within the ventral wall of the cranium and form a lining between the brain and the muscular layers of the oesophagus, and subcutaneous adipocytes are found along the periphery between the muscle and the dermis of the fish [56].

#### 2.4.3. Link between Adipose Tissue and Diabetes

WAT is regarded as an endocrine organ and is mainly comprised of adipocytes, stroma, macrophages, and vasculature. Mature adipocytes express adipokines such as adipsin, adiponectin, and leptin [54], which play a role in the maintenance of beta cell function, breakdown of fatty acids, the regulation of glucose levels, and the regulation of hunger. All these processes are involved in the progression of obesity, which can ultimately result in the development of diabetes mellitus, cardiovascular and liver disease, as well as the predisposition to numerous cancers.

### 2.5. Other Organ Systems Involved in Diabetes

In addition to the organs described, the development and function of other organ systems, such as the skeletal muscle, brain, kidneys, and intestines, are also conserved and all play a role in glycaemic control [26]. The effects of these organ systems in the development of glucose intolerance in individuals with type 2 diabetes range from insulin resistance (brain and skeletal muscle) to increased glucose reabsorption (kidneys) and incretin deficiency/resistance (gastrointestinal tract) [4].

## 3. Wild-Type Zebrafish Larvae as Models for Diabetes and Comorbidities

### 3.1. Diabetes: Causes and Comorbidities

The two main types of diabetes are Diabetes Mellitus type 1 (T1DM) and 2 (T2DM). T1DM is an organ-specific autoimmune disease caused by the destruction of insulin-producing pancreatic beta cells by T-cells [58]. T2DM is a metabolic disease associated with insulin resistance. Hyperglycemia in T2DM is initially accompanied by compensatory hyperinsulinemia, but as the disease progresses, oxidative stress triggers apoptosis of the beta cells, and the amount of insulin produced by the pancreas gradually declines [59]. T2DM is a lifestyle disease often associated with obesity and considered part of the metabolic syndrome. Approximately 90% of diabetics have T2DM [60]. T2DM is accompanied by chronic inflammation and oxidative stress [4,61], and patients often develop non-alcoholic fatty liver disease [62].

Since insulin deficiency is the cause of hyperglycemia in T1DM, the most common treatment for this form of the disease is injection of insulin. In contrast, there are multiple proven and potential drug targets for T2DM. Antidiabetic drugs for T2DM can directly target hyperglycemia by promoting insulin release from beta cells in the early stages of the disease by improving insulin sensitivity of target cells through various pathways, or by slowing down carbohydrate digestion after a meal. T2DM patients can also improve their health through reduction of obesity, inflammation, and oxidative stress [60,61].

Methods using wild-type zebrafish larvae to monitor direct and indirect antidiabetic effects are discussed in the next sections and are summarized in Table 1 and Table 2.

### 3.2. Assessment of Pancreatic Function in Wild-Type Zebrafish Larvae

Pancreatic beta cell destruction is the primary cause of T1DM and a consequence of the progression of T2DM. The toxic glucose analogue alloxan is well known for its use in rodent models of diabetes and has been used successfully in wild-type zebrafish larvae [63]. Nam et al. (2015) treated wild-type larvae at 5 dpf with 100 µM alloxan for 15 min, removed alloxan, and observed a significant reduction in islet area at 6 h and severe damage at 8 h. Subsequent staining with 25 µM of the fluorescent analogue 2-NBDG for one hour enabled visualization and measurement of the pancreatic islet area and intensity using fluorescence microscopy and image analysis software. This method was used to demonstrate how a coffee extract and compounds found in the extract were able to cause regeneration of alloxan damaged pancreatic islets. The results were confirmed in a transgenic zebrafish model that expresses green fluorescence protein (GFP) in pancreatic beta cells. This method, which was used to investigate recovery after alloxan treatment, can be adapted to identify compounds with potential to protect islet cells by exposing the larvae to test samples first and then to alloxan [63].

For wild-type larvae, an alternative method to visualize the pancreas is to use fluorescently conjugated antibodies raised against insulin or glucagon [18]. This is discussed further under oxidative stress. Although Kulkarni, et al. (2018) used this approach as a pancreatic marker, it could potentially be used to quantify the amount of insulin and/or glucagon in the pancreas [18].

### 3.3. Monitoring Glucose Uptake in Wild-Type Zebrafish Larvae

Glucose transporters (GLUTs) in zebrafish have similar structure and tissue distribution as in mammals [76,77]. The fluorescent glucose analogues 2-NBDG and GB2-Cy3 are both known substrates for GLUTs and have been used to monitor glucose uptake in zebrafish larvae. Lee, et al. (2013) illustrated how the insulin mimetic, emodin, could increase 2-NBDG uptake in 72 hpf wild-type larvae. A dose- and time- dependent uptake of 2-NBDG was observed, and 600 µM 2-NBDG for 3 h was selected for further validation of the method. The most sensitive and convenient endpoint proved to be quantification of the increase in green fluorescence in the larval eye, which expresses a high density of GLUTs. The increase in 2-NBDG uptake could be inhibited by the glucose transporter inhibitor 4,6-O-ethylidene-a-d-glucose (4,6 EDG) and reduced in the presence of excess d-glucose to illustrate that 2-NBDG uptake in the zebrafish occurs via GLUTs in a manner similar to that of glucose in mammals. Quantification of 2-NBDG uptake was also possible after lysing the larvae and measuring fluorescence on a plate reader [64], providing a simple method of quantification for laboratories without fluorescence imaging options. 6-NBDG uptake yielded much lower fluorescence intensity and is therefore not a feasible option. Refer to Table 1 footnote for potential pitfalls with emodin as positive control.

Some disadvantages like sensitivity and photobleaching associated with 2-NBDG may be overcome by using newer glucose analogues [22]. Using the method described by Park, et al. (2014) revealed a dose-dependent uptake and much higher signal-to-noise ratio for GB2-Cy3 tested at 5–80 µM, compared to 600 µM 2-NBDG required to provide a quantifiable signal. The GB2-Cy3 signal could also be quantified in the larval eye through imaging or through fluorimetry in lysed larvae [65].

### 3.4. Methods to Assess Glucose Metabolism in Wild-Type Zebrafish Larvae

The pancreas of zebrafish closely resembles that of humans in terms of cell types as well as hormone and digestive enzyme secretion. Regulation of zebrafish genes for insulin, glucagon, and other proteins involved in glucose metabolism has also been shown to be similar in the two species [68]. Increases in endogenous glucose levels in zebrafish larvae are associated with increased expression of the regulatory gluconeogenic enzyme PEPCK [67]. The major regulatory mechanism for this enzyme is activation of expression by cortisone and glucagon, and inhibition by insulin to regulate blood glucose levels. Inhibition of glucagon receptor signaling has been shown to lead to a reduction in PEPCK levels, which in turn improved insulin sensitivity and reduced blood glucose levels in mice with severe insulin resistance [78]. In addition to this, hyperglucagonemia and excessive gluconeogenesis is present in all forms of diabetes [79]. Elo, et al. (2007) used quantitative RT-PCR to show that PEPCK expression is activated in zebrafish larvae exposed to dexamethasone and cAMP at 96 hpf. When larvae were exposed to high glucose concentrations, insulin expression was increased, and PEPCK expression decreased. Metformin, glipizide, and rosiglitazone were capable of returning PEPCK expression levels to control levels. PEPCK can therefore be used as a convenient indicator of glucose homeostasis or dysregulation in very small organisms such as zebrafish larvae where blood extraction is not an option [68].

Direct measurement of in vivo glucose levels in larvae can also be used as an indicator of glucose homeostasis, reflecting the net activity of glycolysis and gluconeogenesis [66,67]. Wild-type larvae can be exposed to treatments, homogenized and glucose measured in the homogenates. Sensitivity is increased by combining 10 larvae per data point and glucose detection performed using a fluorescence-based enzymatic assay. Ten micromolar isoprenaline significantly increased larval glucose levels (i.e., increased gluconeogenesis), and metformin between 50 and 250 µM suppressed this increase in a dose dependent manner [66]. These authors used transgenic zebrafish with a luciferase luminescent PEPCK reporter to illustrate that the effects of isoprenaline and metformin were associated with increased- and decreased PEPCK expression, respectively.

### 3.5. Induction and Monitoring Oxidative Stress in Wild-Type Zebrafish Larvae

Oxidants, reactive oxygen species (ROS) and nitrogen species (NOS), are produced during normal metabolic processes that play essential roles in immunity, cell signaling, and apoptosis. ROS are unstable and reactive molecules that can induce damage to DNA, RNA, lipids, and proteins [80]. Cellular homeostasis is maintained by enzymatic and non-enzymatic antioxidants that neutralize the toxic effects of ROS and NOS. This includes catalase, superoxide dismutase, glutathione reductase, glutathione peroxidase, oxidized glutathione, reduced glutathione, and glutathione S-transferase. When the balance between oxidant and antioxidant is disordered, oxidative stress occurs [81,82].

Studies have highlighted the link between diabetes and oxidative stress by measuring oxidative stress biomarkers present in diabetic patients and rodents. Oxidative biomarkers can be investigated using genetic and biochemical methods.

In a study by Kulkarni, et al. (2018), transgenic nitroreductase-expressing zebrafish were induced with metronidazole (MTZ) to model ROS generation, and the endpoint was determined using CellROX Green in 106 hpf larvae. Oxidative stress induced in the pancreas by MTZ was shown to be dose- and time-dependent. The addition of the antioxidant, *N*-*acetyl*-l-cysteine (NAC), was shown to effectively reduce MTZ-induced ROS at 1- and 6-h treatments, thus exhibiting a protective effect. After staining with CellROX Green, the larvae were fixed and stained with anti-insulin, anti-glucagon, and anti-caspase-3 antibodies, and the DNA was stained with TO-PRO3. The larvae were mounted onto slides and analyzed using confocal microscopy. Pancreatic beta cells are sensitive to excessive levels of ROS due to their low levels of endogenous antioxidant enzymes. Immunofluorescent staining with CellROX and anti-caspase-3 enabled the co-localization and quantification of ROS and apoptosis in the pancreas. This method utilized transgenic larvae but can be adapted for use with wild-type larvae [18].

Zebrafish models for hyperglycemia-stimulated ROS production can also be utilized to determine the protective effect of compounds against oxidative stress. Kim, et al. (2015) demonstrated that phlorotannins (6,6-bieckol, phloroeckol, dieckol, and phlorofucofuroeckol) isolated from *Ecklonia cava* (brown algae) inhibited the production of ROS. The production of ROS was detected using a fluorescent probe dye, 2,7-dichlorodihydrofluorescin diacetate (DCFH-DA). Embryos at 7–9 hpf were pretreated with the test samples (20 µM) for 24 h, and subsequently, 150 mM glucose was added. At 4 dpf, the larvae were treated with the fluorescent probe dye (20 µg/mL) for 1 h. DCFH-DA is deacetylated intracellularly and oxidized by cellular peroxides to fluorescent dichlorofluorescein (DCF). The larvae were anesthetized and the fluorescence observed using fluorescence microscopy and quantified using appropriate analysis software. The study found that one phlorotannin, dieckol, produced a protective effect against oxidative stress. This compound was compared to resveratrol, known for its antioxidant and anti-diabetic effects. A reduction of ROS production was seen for both dieckol (10 and 20 µM) and resveratrol (20 µM). The study also investigated the production of NOS induced by oxidative stress using a fluorescent probe, diaminofluorophore 4-amino-5-methylamino-2′,7′-difluorofluorescein diacetate (DAF-FM DA). Larvae (4 dpf) were treated with DAF-FM DA (5 µM) and incubated for 2 h. The fluorescence was measured using a process similar to that used for ROS production. The study showed that pre-treatment with dieckol and resveratrol decreased the production of NO at 10 and 20 µM concentrations [72].

The fluorescent probe, DCFH-DA, is used in numerous studies to investigate the effect of teratogen induced oxidative stress on development [74] as well as oxidative stress due to pesticide exposure [83]. Gene expression analysis was also performed by quantitative RT-PCR for glutamate-cysteine ligase (*gclc*), Cu/Zn-superoxide dismutase (*sod1*), glutathione S-transferase pi 1 (*gstp1*), and catalase (*cat*) to determine enzymatic activities [74]. Another study by Sapp, et al. (2014) used quantitative RT-PCR to show the upregulation of oxidative stress genes (*gpx* and *irf2a*). Wild-type larvae were immersed in fructose medium from 5–7 dpf and sacrificed. RNA extraction, cDNA synthesis, and quantitative RT-PCR were performed on pooled samples containing eight larvae [75].

A study by Walker, et al. (2012) utilized DCFH-DA to detect ROS production in response to known oxidative stressors (hydrogen peroxide, arsenic trioxide, and phenethyl isothiocyanate). Zebrafish embryos (4 hpf) were pre-treated for 2 h with NAC or a vehicle control and subsequently exposed to the ROS stressors for 30 min. The embryos were then stained with DCFH-DA and quantified using fluorimetry. The study showed that NAC inhibited the production of ROS [73].

### 3.6. Induction and Monitoring Inflammation in Wild-Type Zebrafish Larvae

Inflammation is triggered by numerous stimuli (microbiological, physical, and chemical) that lead to the activation of the immune system and the recruitment of immune cells. This is accompanied by the production of inflammatory mediators, such as pro-inflammatory cytokines (interleukin-6 [IL-6], IL-8, IL-1β, granulocyte-macrophage colony-stimulating factor, and tumor necrosis factor-α [TNF-α]), chemokines (prostaglandin E2 [PGE2] and nitric oxide [NO]), and mediating enzymes (cyclooxygenase-2 [COX-2], matrix metalloproteinases-3 [MMP-3], MMMP-13, and inducible nitric oxide synthase [iNOS]) [84].

Inflammation and oxidative stress pathways are interlinked, forming a positive feedback cycle. The recruitment of immune cells and secretion of cytokines and chemokines leads to the production of ROS and NOS. During diabetes mellitus, chronic inflammation leads to prolonged overproduction of ROS, causing tissue damage [80,85]. Induction of inflammation in wild-type zebrafish larvae has been achieved through exposure to high concentrations of glucose or fructose, as described below.

In addition to activation of ROS, pro-inflammatory enzymes (iNOS and COX-2) are also induced by high glucose treatment. Kim, et al. (2015) investigated ROS/NO production (described in the previous section) and the influence of dieckol and resveratrol on the expression of iNOS and COX-2. The effect was determined using homogenized zebrafish larvae (containing 50 µg of protein) and western blotting to determine iNOS, COX-2, and GAPDH (internal control) expression. Detection was done using an enhanced chemiluminescence western blotting detection kit and exposure to X-ray film. The study found that dieckol and resveratrol treatment (20 µM) reduced expression of iNOS and COX-2 [72].

Fructose treatment for 48 h can induce hepatic steatosis in wild-type zebrafish larvae, causing activation of inflammatory pathways. The expression of inflammatory genes (*nfkb*, *irf2a*, *tnfa*) was investigated by quantitative RT-PCR, which showed increased expression after treatment [75].

### 3.7. Modelling Obesity in Wild-Type Zebrafish Larvae

Obesity is a worldwide public health priority and is characterized by increased fat storage in WAT. To date, the main cause of obesity remains the quality and quantity of food that is consumed by individuals, although evidence exists that exposure to obesogens, a class of endocrine-disrupting chemicals, during early life, also affects adipogenesis and lipid metabolism [86,87]. It is a well-known fact that a reduction in body weight can alleviate the symptoms of T2DM in obese diabetics.

Obesity is most commonly studied in animals using a diet-induced model. Zebrafish larvae become self-feeding around 5–6 dpf, at which stage lipids are present in many tissues [88]. Adipogenesis starts at 8 dpf near the pancreas in the visceral cavity; however, WAT is not detected in zebrafish embryos and early larvae until 12–15 dpf, but these stages have been shown to be susceptible to effectors of fat metabolism. The first diet-induced model of obesity in zebrafish was established by Oka, et al. (2010) [89]. Other models, such as those established by Meguro, et al. (2015), Forn-Cuni, et al. (2015), Turola, et al. (2015), David, et al. (2016), Zang, et al. (2017), and Landgraf, et al. (2017), followed a similar approach and fed zebrafish high caloric diets for periods ranging from 6–24 weeks and even eight months [90,91,92,93,94,95]. Phenotypic observations from these studies support the use of zebrafish to model obesity in a diet-induced manner. However, these models lack uniformity in that they all differ in the diet regimens used, and thus a more standardized model is needed to limit the variations in energy intake, body composition, and adipose tissue deposition that may arise [88].

Another potential downside of the models mentioned above is the number of weeks or months for which zebrafish were fed a high caloric diet. This could also tie in with the 3 R’s of animal-related research, more specifically refinement, in which models are refined to minimize potential pain, suffering, or distress experienced. Therefore, models in which shorter periods can be used would be ideal, not only for animal welfare, but also to speed up the drug discovery process. Studies by Clifton, et al. (2010), Tingaud-Sequeira, et al. (2011), and Zhou, et al. (2015), where early stage larvae were used, may serve this purpose [86,96,97].

In the study by Clifton, et al. (2010), zebrafish larvae at 5 dpf were exposed to novel inhibitors of dietary lipid absorption overnight and then incubated in fluorescent lipid reporters such as PED-6 (phospholipid), NBD-cholesterol, BODIPY-C5 (short-chain fatty acid analogue), and BODIPY-C16 (long-chain fatty acid analogue) for 5–6 h [96]. Treatment with ezetimibe resulted in an expected decrease in NBD-cholesterol metabolism. However, an unexpected decrease in PED-6 and BODIPY-C16 metabolism was also observed, but not for BODIPY-C5. Oil Red O staining of larvae at 6 dpf was also used to illustrate intestinal lipid absorption following feeding with 1% egg yolk and a marked reduction in lipid absorption with the novel inhibitors (25 µM), and ezetimibe (50 µM) was observed [96].

Various other dyes can be used as alternatives to Oil Red O, since it is associated with a high incidence of photobleaching as well as strong background staining and difficulty in the quantification of actual lipid content [86,97]. These dyes include Sudan, Nile Red, and LipidGreen.

Most commonly, the larval zebrafish models utilize a certain percentage of egg-yolk as the inducer of obesity [86,97]. However, alternatives to this include the use of heavy whipping cream [88,98].

As opposed to the norm, Tingaud-Sequeira, et al. (2011) established a zebrafish obesogenic assay based on the standard length of larvae. The zebrafish obesogenic assay entails the feeding of larvae with either a standard diet or hard-boiled chicken egg yolk high-fat diet with total fat contents of 10% and 55%, respectively, for 1 day. The authors found that, for optimal results to be obtained with Nile Red staining, the larvae had to have a standard length of between 7.5 and 9 mm. Nile Red staining was done by incubating the live larvae in a 5 µg/mL solution of Nile Red for 30 min at 28 °C. Furthermore, to determine the whole-body triacylglycerol content, Tingaud-Sequeira, et al. (2011) starved the larvae for 24 h prior to Nile Red staining and euthanasia. Whole larvae were homogenized and prepared for a commercial colourimetric microassay. Results obtained for treatment with rosiglitazone, phenylephrine, and tributyltin chloride indicated that this assay was suitable for the screening of drugs for their ability to impair fat storage and mobilization [86].

The use of zebrafish to screen drugs for their modulatory effect on adipocyte number and lipid accumulation remains a promising tool. However, it is worth noting that zebrafish rely on thyroid-driven transcriptional mechanisms to generate heat, and secretion of lipoprotein particles by female adipocytes is regulated by oestrogen. The latter is a process known as vitellogenesis, which is not found in mammals, where lipids are directed towards the ovaries [23].

A summary of the wild-type zebrafish larvae obesity models discussed above is presented in Table 3.

### 3.8. Wound Healing in Wild-Type Zebrafish Larvae

Impaired wound healing is one of the major secondary complications of diabetes and can result in limb amputation and disability. Lower limb amputation is the most common complication that results from nonhealing wounds in diabetic individuals. Annually, more than 200 billion dollars is generated in healthcare costs from diabetes-related amputations. This, together with the high mortality rates associated with the operation, makes amputations more expensive and more dangerous than certain types of cancer [99].

Diabetes-related factors that affect the wound healing process include the vascular, neuropathologic, cellular, immune function, and biochemical abnormalities. Diabetic individuals are known to suffer from poor glucose control, leading to hyperglycemia, which ultimately impairs wound healing. This is achieved by the alteration of Na^+^/K^+^ ATPase activity as well as increased production of both protein kinase C (PKC) and advanced glycation end products (AGEs) [100,101,102]. PKC modulates neovascularization and cell growth in vascular cells, whereas AGEs lead to increased permeability of blood vessels as well as thickened and inelastic vessel walls. AGEs also alter gene expression and other cellular aspects by increasing oxidative stress [102,103].

Normal wound repair consists of four distinct, yet overlapping, stages including homeostasis, inflammation, proliferation, and remodelling [99,104]. In a hyperglycemic environment, granulocytes, keratinocytes, and fibroblasts show impaired functioning. This results in altered migration/chemotaxis and adherence of neutrophils as well as a decrease in the release of cytokines and growth factors such as vascular endothelial growth factor (VEGF). Furthermore, impaired keratinocyte and fibroblast functioning also compromises collagen synthesis, resulting in inflexible and fragile collagen [99,102].

Zebrafish can regenerate many tissues and organs. Wounds show rapid re-epithelialization (within hours), independent of coagulation and inflammation. Granulation-like tissue is formed and later cleared, resulting in minimal scar formation [104]. Healing processes occur sequentially in zebrafish as opposed to the overlapping phases in mammals. This allows for better identification of direct and indirect effects caused by chemical or genetic manipulation. It also provides an opportunity to perform high-throughput small-molecule drug screens [104,105].

Adult zebrafish of 3–6 months [106,107,108,109] or 6–12 months [104] are the most commonly used in studies related to wound healing. However, all these studies use the fin regeneration model of zebrafish due to the remarkable ability of zebrafish to regenerate their fins after amputation. The organization of the larvae fin fold, at 2 dpf, is composed of a middle mesenchymal layer containing fibroblast-like cells, actinotrichia, and nerves. This layer is surrounded by two epidermal layers containing keratinocytes and an underlying basement membrane [110,111]. Fin regeneration is comprised of three stages including wound healing (wound closes within 12 h post amputation (hpa)), proliferative blastema formation (mesenchymal cells aggregate at the plane of amputation between 1–3 days post amputation (dpa)), and cell differentiation (fin outgrowth occurs between 3–14 dpa) [109].

Studies by Niethammer, et al. (2009), Yoo, et al. (2010), LeBert, et al. (2015), Miskolci, et al. (2019), and Beckman (2019) all demonstrated that larval zebrafish of 2–4 dpf can also be used for the caudal fin regeneration model of wound healing [112,113,114,115,116]. However, none of these models have studied the direct effect of diabetes on wound healing, but rather have focused on the damage-induced migration of neutrophils, production of reactive oxygen species, onset of inflammation, and general re-growth of the caudal fin after amputation.

The migration of neutrophils to a wound site is an essential inflammatory response for the prevention of infection by microorganisms and aid in the closure of the wound [112,117]. Niethammer, et al. (2009) found that, compared to the millions of neutrophils that migrate to a wound site in humans, only a mere 20–30 neutrophils are found to have migrated to a wound site in the ventral tail fin of four-day old zebrafish larvae at 3 h post amputation (hpa). The migration was deemed to have been in response to the generation of a hydrogen peroxide (H_2_O_2_) gradient by the NADPH oxidase, Duox, upon wounding. The neutrophils were fluorescently labelled with leukocyte-specific tags, *mpo::GFP* and *lysC::DsRED2* [112].

Neutrophils in 2–3 dpf wild-type larvae can also be detected by Sudan black staining as described by Yoo, et al. (2012) [118]. In this method, larvae were fixed for 1.5 h in 4% formaldehyde at room temperature, rinsed with PBS, and then stained with 0.03% Sudan black. Extensive washing with 70% ethanol, followed by rehydration in PBS with 0.1% Tween 20, was done prior to microscopic observation [113,114,118].

In the study by Beckman (2019), the caudal fin of a larvae at 3 dpf was amputated posterior to the notochord under anaesthesia with tricaine. Bright-field microscopy with z-stacking was the preferred method to image the growth of the amputated caudal fin at 1.5 hpa and 1, 2, and 3 dpa. Results indicated that the fin was almost completely regenerated by the third day post amputation [116]. The same observations were made by Miskolci, et al. (2019).

Since neutrophils are the first type of cells to migrate to a wound site, the presence of these cells in the caudal fin, pre- and post-wounding, was also detected by Beckman (2019). This was done using a fluorescent rabbit polyclonal anti-mpx antibody (1:100) overnight. Results obtained using fluorescence microscopy indicated that there is a marked increase in the number of neutrophils that migrate to the wound site upon amputation and that at 3 dpa, the number returns to pre-wounding levels [116].

### 3.9. Non-Alcoholic Fatty Liver Disease

Non-alcoholic fatty liver disease (NAFLD) is commonly characterized by excess fat build-up in the liver, leading to steatosis, steatohepatitis, and fibrosis, regardless of the patient’s alcohol consumption level. Non-alcoholic fatty liver disease and diabetes (especially type 2 diabetes (T2DM)) often co-exist and act synergistically to initiate the onset of other adverse pathologies. Current models of NAFLD aim to reduce modifiable metabolic risks through controlling glycaemia, optimizing weight loss, and screening for further complications to minimize the risk of hepatic decompensation [62,119].

The implementation of more sophisticated techniques over the years has provided a means to better understand the mechanism of NAFLD and in turn how to manage NAFLD. These techniques incorporate in vivo animal models like zebrafish larvae, along with fluorescent dyes that offer both quantitative and qualitative information on the areas in question. In a study by Sapp, et al. (2014), zebrafish larvae (treated from 5–7 dpf) were immersed in 4% fructose to induce non-alcoholic steatohepatitis and in glucose for calorie-matched controls. After appropriate fixing and staining with Oil Red O, results indicated that lipid accumulation, mitochondrial abnormalities, and endoplasmic reticulum (ER) defects each play a role in NAFLD. Moreover, fructose-treated zebrafish larvae displayed activation of inflammatory, oxidative stress and lipogenic genes through quantitative PCR methods, proving zebrafish to be an appropriate model for NAFLD and diabetes studies [75]. Chen, et al. (2018) and Ahmad, et al. (2019) emphasized how dietary composition has an important impact on NAFLD in zebrafish larvae; indicating that zebrafish larvae fed with high cholesterol (HC), high fructose (HF), and extra feeding (EF) diets for 10 days displayed varying degrees of hepatic steatosis, where EF had the most detrimental steatosis effect [120,121]. Ma, et al. (2019) also promoted zebrafish as an attractive model for NAFLD related studies, as it provided a quick, multiple index model to closely address all the features of NAFLD [122]. Khneizer, et al. (2020) proposed therapeutic agents such as vitamin E, pioglitazone, and metformin to help the diabetes mellitus and NAFLD duo.

## 4. Advantages, Disadvantages, Shortcomings, and Suggestions

The main advantages of using wild-type zebrafish larvae for drug screening revolve around ethical considerations (three R’s) and easy access and care of the animals (compared to genetically modified models). However, using animals to model human disease will always have certain limitations, and zebrafish are no exception. It is important to recognize the limitations of each animal model and interpret any data obtained from them within those limitations.

In general, the methods used for toxicity and antidiabetic testing using zebrafish larvae most frequently make use of bright field microscopy, fluorescent dyes in conjunction with fluorimetry or imaging techniques, western blotting, and quantitative RT-PCR to monitor behavioral or morphological changes as well as protein and gene expression levels.

Wild-type zebrafish larvae can be used successfully in the assessment of acute and developmental toxicity, hepatotoxicity, and cardiotoxicity, with existing methods mostly utilizing microscopic evaluation of phenotypic features. This approach is not conducive to high throughput screening, but can be useful for smaller sample numbers in laboratories where limited facilities are available. Fluorescent dyes have been used to detect signs of steatosis, oxidative stress, and mitochondrial dysfunction in larval zebrafish models of NAFLD. It is quite plausible that the same methods could be used to screen for DILI and this could, at the same time, improve throughput.

Methods modelling diabetes in wild-type larvae seem very promising. Although screening for inhibition of complex carbohydrate digesting enzymes has never been reported and is unlikely to work, in vitro enzymatic assays are simple to perform as an alternative. Induction of insulin resistance and monitoring aspects involving glucose uptake and metabolism can be performed in larvae using combinations of biochemical assays, imaging techniques, western blotting, and quantitative RT-PCR. The relatively easy method of measuring larval glucose levels to monitor gluconeogenesis [66,67] without the need for specialized equipment could potentially be used in many laboratories where high-end equipment is not available. It should be feasible to achieve a relatively high throughput by inducing insulin resistance in larvae through exposure to glucocorticoids and cAMP and then monitoring larval glucose in parallel with the uptake of a fluorescent glucose analogue in response to insulin mimetics [64,65]. Through this approach, it should be possible to identify compounds with potential to reduce hepatic glucose output, improve peripheral glucose uptake, and reduce insulin resistance.

Oxidative stress and inflammation are closely interlinked, and induction in wild-type larvae is achievable through exposure to high concentrations of glucose or fructose. These responses seem to correlate quite well with oxidative stress and inflammatory responses in humans. Although not many publications have appeared on this topic, fluorescent dyes such as CellRox Green have been used in conjunction with fluorescence imaging or fluorometry, while quantitative RT-PCR has mostly been used to monitor expression levels of enzymes such as iNOS and COX-2. Interestingly, no reports were found where larval cytokine levels were quantified with ELISA. This should be feasible and easy to do using larval homogenates.

Wound healing assays in zebrafish are mostly done on adults, and there is scope for improvement and expansion of the few methods that have been reported on larvae. The major events of mammalian skin repair have been shown to be present in zebrafish and thus make zebrafish an ideal candidate as a skin animal model. Limitations to the use of zebrafish include the presence of scales in adult fish and a lack of appendages and an epidermal barrier. However, systemic drug screening is made easier, due to the lack of the epidermal barrier, as compounds can simply be added to the water [123]. Furthermore, the molecular and histological (presence of keratinocytes and fibroblasts) similarities between zebrafish and human skin composition increases the attractiveness of using zebrafish to understand underlying mechanisms of skin disease, wound healing, and regeneration [124].

## 5. Conclusions

The advantages of genetically modified zebrafish models in antidiabetic drug discovery cannot be contested. However, the cost to purchase and maintain them and the need for multiple strains that model the myriad of different antidiabetic drug targets renders their use extremely challenging and often impractical to many research laboratories. In contrast, wild-type zebrafish are easy to maintain and represent a suitable alternative to overcome such limitations. Many published reports exist demonstrating the successful application of wild-type zebrafish, including their larvae, to model diabetes and common comorbidities like obesity, inflammation, and oxidative stress. Given the integral role of animal models in the drug discovery pipeline, wild-type zebrafish larvae constitute a practical alternative to other more costly vertebrate options. An integrated approach combining target directed in vitro screening methodologies, such as enzyme and cell-based assays to identify/confirm specific target efficacy, followed by the appropriate zebrafish model(s), can provide a powerful platform for preclinical drug discovery. Furthermore, this approach can also be used to assess toxicity risks, a major contributory factor in drug attrition. Expanding the platform to include modern in vitro technologies such as cellomics can further improve the accuracy of risk assessment. Using wild-type zebrafish larvae as an alternative to mammalian models of diabetes may not only reduce research cost and increase throughput, but also contribute significantly to the three Rs principle of humane animal experimentation, while simultaneously elevating the pace of antidiabetic drug discovery.

## Figures and Tables

**Table 1:** Existing methods suitable for antidiabetic activity testing using wild-type zebrafish larvae.

Target	Larval Age	Treatment or Positive Controls	Detection	References
Pancreatic function	5 dpf	Alloxan to destroy beta cells	Fluorescent staining with 2-NBDG to measure islet size	[63]
Glucose uptake	3 dpf	Insulin mimetics, e.g., emodin *	2-NBDG or GB2-Cy3 fluorescence in the eye or flourimetry on homogenized larvae	[64,65]
Glucose production (gluconeogenesis)	From 4 dpf when yolk depletes	Gluconeogenesis stimulated with glucocorticoids, cAMP, isoprenaline	Glucose oxidase assay with sensitive fluorescence detection	[66,67]
PEPCK expression levels (gluconeogenesis)	4 dpf	PEPCK levels increased with glucocorticoids and cAMP	Quantitative RT-PCR	[68]

* Emodin toxicity has been reported [69], and its intrinsic fluorescence properties [70] may interfere if used in conjunction with certain fluorescent dyes. It may be advisable to replace it with a positive control such as ampkinone, a small-molecule AMP-Activated Protein Kinase activator with antidiabetic and anti-obesity activity [71].

**Table 2:** Methods suitable for testing anti-inflammatory- and reduction of oxidative stress activity using wild-type zebrafish larvae.

Target	Larval Age	Treatment or Positive Controls	Detection	References
ROS levels	4–106 hpf	Metronidazole (inducer of ROS) or high glucose/fructose concentration or H_2_O_2_ Resveratrol or *N*-*acetyl*-l-cysteine as inhibitors	Fluorimetry or fluorescence microscopy after staining with CellROX Green or DCFH-DA Immunofluorescent staining of insulin or glucagon can be used to locate pancreas	[18,72,73]
NOS levels	4 dpf	As above	As above, but staining with DAF-FM DA	[72]
Expression levels of genes involved in inflammation and oxidative stress (including iNOS, COX-2 and others)	5–7 dpf	High glucose/fructose concentrations	Quantitative RT-PCR	[72,74,75]

**Table 3:** Methods suitable for modelling obesity using wild-type zebrafish larvae.

Target	Larval Age	Treatment or Positive Controls	Detection	References
Inhibition of dietary lipid absorption	4–6 dpf	Ezetimibe used as inhibitor	Fluorescently stained with PED-6, NBD-cholesterol, BODIPY-C5, BODIPY-C16, and BODIPY-C12	[96,98]
Intestinal lipid absorption, hyperlipidemia, lipid accumulation in vasculature and whole-larval TAG	5–6 dpf	Fed with 1% egg yolkEzetimibe, lovastatin and simvastatin used as inhibitors	Oil Red O staining	[96,97]
			Lipid Green staining	[87]
	6 dpf	Heavy whipping cream	Oil Red O staining	[98]
	Size dependent (standard length of 7.5–9 mm)	Hard-boiled chicken egg yolk (55% total fat content)Rosiglitazone, phenylephrine and tributyltin chloride used as inhibitors	Nile Red staining	[86]

## Data Availability

No new data were created or analyzed in this study. Data sharing is not applicable to this article.

## References

[1] Williams R., Karuranga S., Malanda B., Saeedi P., Basit A., Besancon S., Bommer C., Esteghamati A., Ogurtsova K., Zhang P. (2020). Global and regional estimates and projections of diabetes-related health expenditure: Results from the International Diabetes Federation Diabetes Atlas, 9th edition. Diabetes Res. Clin. Pract..

[2] Williams R., Colagiuri S., Chan J., Gregg E., Ke C., Lim L.-L., Yang X. (2019). IDF Diabetes Atlas.

[3] International Diabetes Federation IDF DIABETES ATLAS 9th Edition 2019. https://diabetesatlas.org/en/.

[4] Defronzo R.A. (2009). Banting Lecture. From the triumvirate to the ominous octet: A new paradigm for the treatment of type 2 diabetes mellitus. Diabetes.

[5] Chaudhury A., Duvoor C., Reddy Dendi V.S., Kraleti S., Chada A., Ravilla R., Marco A., Shekhawat N.S., Montales M.T., Kuriakose K. (2017). Clinical Review of Antidiabetic Drugs: Implications for Type 2 Diabetes Mellitus Management. Front. Endocrinol. (Lausanne).

[6] Blaslov K., Naranda F.S., Kruljac I., Renar I.P. (2018). Treatment approach to type 2 diabetes: Past, present and future. World J. Diabetes.

[7] Vikram A., Tripathi D.N., Kumar A., Singh S. (2014). Oxidative stress and inflammation in diabetic complications. Int. J. Endocrinol.

[8] Pickering R.J., Rosado C.J., Sharma A., Buksh S., Tate M., de Haan J.B. (2018). Recent novel approaches to limit oxidative stress and inflammation in diabetic complications. Clin. Transl. Immunol..

[9] Kleinert M., Clemmensen C., Hofmann S.M., Moore M.C., Renner S., Woods S.C., Huypens P., Beckers J., de Angelis M.H., Schurmann A. (2018). Animal models of obesity and diabetes mellitus. Nat. Rev. Endocrinol..

[10] Al-Awar A., Kupai K., Veszelka M., Szucs G., Attieh Z., Murlasits Z., Torok S., Posa A., Varga C. (2016). Experimental Diabetes Mellitus in Different Animal Models. J. Diabetes Res..

[11] Van de Venter M., Boukes G., Venables L., Dambuza N., Koekemoer T. (2017). The Need for an Integrated, Multi-target In vitro Anti-diabetic Screening Platform. Drug Discovery from Herbs: Approaches and Applications.

[12] Sneddon L.U., Halsey L.G., Bury N.R. (2017). Considering aspects of the 3Rs principles within experimental animal biology. J. Exp. Biol..

[13] Teame T., Zhang Z., Ran C., Zhang H., Yang Y., Ding Q., Xie M., Gao C., Ye Y., Duan M. (2019). The use of zebrafish (*Danio rerio*) as biomedical models. Anim Front..

[14] Nishimura Y., Inoue A., Sasagawa S., Koiwa J., Kawaguchi K., Kawase R., Maruyama T., Kim S., Tanaka T. (2016). Using zebrafish in systems toxicology for developmental toxicity testing. Congenit Anom (Kyoto).

[15] Fleming A., Alderton W.K. (2013). Zebrafish in pharmaceutical industry research: Finding the best fit. Drug Discov. Today Dis. Models.

[16] MacRae C.A., Peterson R.T. (2015). Zebrafish as tools for drug discovery. Nat. Rev. Drug Discov..

[17] Strahle U., Scholz S., Geisler R., Greiner P., Hollert H., Rastegar S., Schumacher A., Selderslaghs I., Weiss C., Witters H. (2012). Zebrafish embryos as an alternative to animal experiments—A commentary on the definition of the onset of protected life stages in animal welfare regulations. Reprod. Toxicol..

[18] Kulkarni A.A., Conteh A.M., Sorrell C.A., Mirmira A., Tersey S.A., Mirmira R.G., Linnemann A.K., Anderson R.M. (2018). An In Vivo Zebrafish Model for Interrogating ROS-Mediated Pancreatic beta-Cell Injury, Response, and Prevention. Oxid. Med. Cell. Longev..

[19] Kinkel M.D., Prince V.E. (2009). On the diabetic menu: Zebrafish as a model for pancreas development and function. Bioessays.

[20] Jorgens K., Hillebrands J.L., Hammes H.P., Kroll J. (2012). Zebrafish: A model for understanding diabetic complications. Exp. Clin. Endocrinol. Diabetes.

[21] Seth A., Stemple D.L., Barroso I. (2013). The emerging use of zebrafish to model metabolic disease. Dis Model. Mech.

[22] Tabassum N., Tai H., Jung D.W., Williams D.R. (2015). Fishing for Nature’s Hits: Establishment of the Zebrafish as a Model for Screening Antidiabetic Natural Products. Evid. Based Complement. Altern. Med..

[23] Schlegel A., Gut P. (2015). Metabolic insights from zebrafish genetics, physiology, and chemical biology. Cell. Mol. Life Sci..

[24] Gut P., Reischauer S., Stainier D.Y.R., Arnaout R. (2017). Little Fish, Big Data: Zebrafish as a Model for Cardiovascular and Metabolic Disease. Physiol. Rev..

[25] Heckler K., Kroll J. (2017). Zebrafish as a Model for the Study of Microvascular Complications of Diabetes and Their Mechanisms. Int. J. Mol. Sci..

[26] Zang L., Maddison L.A., Chen W. (2018). Zebrafish as a Model for Obesity and Diabetes. Front. Cell Dev. Biol..

[27] Kelly J.R., Benson S.A. (2020). Inconsistent ethical regulation of larval zebrafish in research. J. Fish. Biol..

[28] Hernandez R.E., Galitan L., Cameron J., Goodwin N., Ramakrishnan L. (2018). Delay of Initial Feeding of Zebrafish Larvae Until 8 Days Postfertilization Has No Impact on Survival or Growth Through the Juvenile Stage. Zebrafish.

[29] The Zebrafish Information Network ZFIN: Wild-Type Lines: Summary Listing. https://zfin.org/action/feature/wildtype-list.

[30] Sarmah S., Marrs J.A. (2016). Zebrafish as a Vertebrate Model System to Evaluate Effects of Environmental Toxicants on Cardiac Development and Function. Int. J. Mol. Sci..

[31] Brown D.R., Samsa L.A., Qian L., Liu J. (2016). Advances in the Study of Heart Development and Disease Using Zebrafish. J. Cardiovasc. Dev. Dis..

[32] Staudt D., Stainier D. (2012). Uncovering the molecular and cellular mechanisms of heart development using the zebrafish. Annu. Rev. Genet..

[33] Glickman N.S., Yelon D. (2002). Cardiac development in zebrafish coordination of form and function. Semin. Cell Dev. Biol..

[34] Bakkers J. (2011). Zebrafish as a model to study cardiac development and human cardiac disease. Cardiovasc. Res..

[35] Ahuja N., Ostwald P., Bark D., Garrity D. (2020). Biomechanical Cues Direct Valvulogenesis. J. Cardiovasc. Dev. Dis..

[36] Shrestha R., Lieberth J., Tillman S., Natalizio J., Bloomekatz J. (2020). Using Zebrafish to Analyze the Genetic and Environmental Etiologies of Congenital Heart Defects. Adv. Exp. Med. Biol..

[37] Scherz P.J., Huisken J., Sahai-Hernandez P., Stainier D.Y. (2008). High-speed imaging of developing heart valves reveals interplay of morphogenesis and function. Development.

[38] Stratmann B., Tschoepe D. (2011). Heart in diabetes: Not only a macrovascular disease. Diabetes Care.

[39] Rosano G.M., Vitale C., Seferovic P. (2017). Heart Failure in Patients with Diabetes Mellitus. Card. Fail. Rev..

[40] Wang S., Miller S.R., Ober E.A., Sadler K.C. (2017). Making It New Again: Insight into Liver Development, Regeneration, and Disease from Zebrafish Research. Curr. Top. Dev. Biol..

[41] Wilkins B.J., Pack M. (2013). Zebrafish models of human liver development and disease. Compr. Physiol..

[42] Goessling W., Sadler K.C. (2015). Zebrafish: An important tool for liver disease research. Gastroenterology.

[43] Wu J., Lu C., Ge S., Mei J., Li X., Guo W. (2020). Igf2bp1 is required for hepatic outgrowth during early liver development in zebrafish. Gene.

[44] Field H.A., Ober E.A., Roeser T., Stainier D.Y. (2003). Formation of the digestive system in zebrafish. I. Liver morphogenesis. Dev. Biol..

[45] Tao T., Peng J. (2009). Liver development in zebrafish (*Danio rerio*). J. Genet. Genom..

[46] Yi X., Yu J., Ma C., Li L., Luo L., Li H., Ruan H., Huang H. (2018). Yap1/Taz are essential for the liver development in zebrafish. Biochem. Biophys. Res. Commun..

[47] Mohamed J., Nazratun Nafizah A.H., Zariyantey A.H., Budin S.B. (2016). Mechanisms of Diabetes-Induced Liver Damage: The role of oxidative stress and inflammation. Sultan Qaboos Univ. Med. J..

[48] Tolman K.G., Fonseca V., Dalpiaz A., Tan M.H. (2007). Spectrum of liver disease in type 2 diabetes and management of patients with diabetes and liver disease. Diabetes Care.

[49] Gnugge L., Meyer D., Driever W. (2004). Pancreas Development in Zebrafish. Methods in Cell Biology.

[50] Li Z., Wen C., Peng J., Korzh V., Gong Z. (2009). Generation of living color transgenic zebrafish to trace somatostatin-expressing cells and endocrine pancreas organization. Differentiation.

[51] Prince V.E., Anderson R.M., Dalgin G. (2017). Zebrafish Pancreas Development and Regeneration: Fishing for Diabetes Therapies. Curr. Top. Dev. Biol..

[52] Field H.A., Dong P.D.S., Beis D., Stainier D.Y.R. (2003). Formation of the digestive system in zebrafish. ii. pancreas morphogenesis. Dev. Biol..

[53] Wendelaar Bonga S.E., Evans D.E. (1993). Endocrinology. The Physiology of Fishes.

[54] Gesta S., Tseng Y.H., Kahn C.R. (2007). Developmental origin of fat: Tracking obesity to its source. Cell.

[55] Minchin J.E., Rawls J.F. (2011). In vivo analysis of white adipose tissue in zebrafish. Methods Cell Biol..

[56] Imrie D., Sadler K.C. (2010). White adipose tissue development in zebrafish is regulated by both developmental time and fish size. Dev. Dyn..

[57] Elemans L.M.H., Cervera I.P., Riley S.E., Wafer R., Fong R., Tandon P., Minchin J.E.N. (2019). Quantitative analyses of adiposity dynamics in zebrafish. Adipocyte.

[58] Wang Z., Xie Z., Lu Q., Chang C., Zhou Z. (2017). Beyond Genetics: What Causes Type 1 Diabetes. Clin. Rev. Allergy Immunol..

[59] Hollander P.A., Kushner P. (2010). Type 2 Diabetes Comorbidities and Treatment Challenges Rationale for DPP-4 Inhibitors. Postgrad. Med..

[60] DeFronzo R.A., Ferrannini E., Groop L., Henry R.R., Herman W.H., Holst J.J., Hu F.B., Kahn C.R., Raz I., Shulman G.I. (2015). Type 2 diabetes mellitus. Nat. Rev. Dis. Primers.

[61] Teodoro J.S., Nunes S., Rolo A.P., Reis F., Palmeira C.M. (2019). Therapeutic Options Targeting Oxidative Stress, Mitochondrial Dysfunction and Inflammation to Hinder the Progression of Vascular Complications of Diabetes. Front. Physiol..

[62] Khneizer G., Rizvi S., Gawrieh S. (2020). Non-alcoholic Fatty Liver Disease and Diabetes Mellitus. Advances in Experimental Medicine and Biology.

[63] Nam Y.H., Hong B.N., Rodriguez I., Ji M.G., Kim K., Kim U.J., Kang T.H. (2015). Synergistic Potentials of Coffee on Injured Pancreatic Islets and Insulin Action via KATP Channel Blocking in Zebrafish. J. Agric. Food Chem..

[64] Lee J., Jung D.W., Kim W.H., Um J.I., Yim S.H., Oh W.K., Williams D.R. (2013). Development of a highly visual, simple, and rapid test for the discovery of novel insulin mimetics in living vertebrates. ACS Chem. Biol..

[65] Park J., Um J.I., Jo A., Lee J., Jung D.W., Williams D.R., Park S.B. (2014). Impact of molecular charge on GLUT-specific cellular uptake of glucose bioprobes and in vivo application of the glucose bioprobe, GB2-Cy3. Chem. Commun..

[66] Gut P., Baeza-Raja B., Andersson O., Hasenkamp L., Hsiao J., Hesselson D., Akassoglou K., Verdin E., Hirschey M.D., Stainier D.Y. (2013). Whole-organism screening for gluconeogenesis identifies activators of fasting metabolism. Nat. Chem. Biol..

[67] Jurczyk A., Roy N., Bajwa R., Gut P., Lipson K., Yang C., Covassin L., Racki W.J., Rossini A.A., Phillips N. (2011). Dynamic glucoregulation and mammalian-like responses to metabolic and developmental disruption in zebrafish. Gen. Comp. Endocrinol..

[68] Elo B., Villano C.M., Govorko D., White L.A. (2007). Larval zebrafish as a model for glucose metabolism: Expression of phosphoenolpyruvate carboxykinase as a marker for exposure to anti-diabetic compounds. J. Mol. Endocrinol..

[69] Dong X., Fu J., Yin X., Cao S., Li X., Lin L., Ni J., Huyiligeqi (2016). Emodin: A Review of its Pharmacology, Toxicity and Pharmacokinetics. Phytother. Res..

[70] Vargas F., Rivas C., Medrano M. (2004). Interaction of emodin, aloe-emodin, and rhein with human serum albumin: A fluorescence spectroscopic study. Toxicol. Mech. Methods.

[71] Oh S., Kim S.J., Hwang J.H., Lee H.Y., Ryu M.J., Park J., Kim S.J., Jo Y.S., Kim Y.K., Lee C.H. (2010). Antidiabetic and antiobesity effects of Ampkinone (6f), a novel small molecule activator of AMP-activated protein kinase. J. Med. Chem..

[72] Kim E.-A., Kang M.-C., Lee J.-H., Kang N., Lee W., Oh J.-Y., Yang H.-W., Lee J.-S., Jeon Y.-J. (2015). Protective effect of marine brown algal polyphenols against oxidative stressed zebrafish with high glucose. RSC Adv..

[73] Walker S.L., Ariga J., Mathias J.R., Coothankandaswamy V., Xie X., Distel M., Koster R.W., Parsons M.J., Bhalla K.N., Saxena M.T. (2012). Automated reporter quantification in vivo: High-throughput screening method for reporter-based assays in zebrafish. PLoS ONE.

[74] Félix L.M., Vidal A.M., Serafim C., Valentim A.M., Antunes L.M., Campos S., Matos M., Monteiro S.M., Coimbra A.M. (2016). Ketamine-induced oxidative stress at different developmental stages of zebrafish (*Danio rerio*) embryos. RSC Adv..

[75] Sapp V., Gaffney L., EauClaire S.F., Matthews R.P. (2014). Fructose leads to hepatic steatosis in zebrafish that is reversed by mechanistic target of rapamycin (mTOR) inhibition. Hepatology.

[76] Tseng Y.C., Chen R.D., Lee J.R., Liu S.T., Lee S.J., Hwang P.P. (2009). Specific expression and regulation of glucose transporters in zebrafish ionocytes. Am. J. Physiol. Regul. Integr. Comp. Physiol..

[77] Jimenez-Amilburu V., Jong-Raadsen S., Bakkers J., Spaink H.P., Marin-Juez R. (2015). GLUT12 deficiency during early development results in heart failure and a diabetic phenotype in zebrafish. J. Endocrinol..

[78] Okamoto H., Cavino K., Na E., Krumm E., Kim S.Y., Cheng X., Murphy A.J., Yancopoulos G.D., Gromada J. (2017). Glucagon receptor inhibition normalizes blood glucose in severe insulin-resistant mice. Proc. Natl. Acad. Sci. USA.

[79] Lee Y.H., Wang M.Y., Yu X.X., Unger R.H. (2016). Glucagon is the key factor in the development of diabetes. Diabetologia.

[80] Mourabit S., Fitzgerald J.A., Ellis R.P., Takesono A., Porteus C.S., Trznadel M., Metz J., Winter M.J., Kudoh T., Tyler C.R. (2019). New insights into organ-specific oxidative stress mechanisms using a novel biosensor zebrafish. Environ. Int..

[81] Gyimah E., Dong X., Qiu W., Zhang Z., Xu H. (2020). Sublethal concentrations of triclosan elicited oxidative stress, DNA damage, and histological alterations in the liver and brain of adult zebrafish. Environ. Sci. Pollut. Res. Int..

[82] Sobhi W., Khenchouche A. (2020). Involvement of Oxidative Stress in Type 1 Diabetes. Am. J. Biomed. Sci. Res..

[83] Velki M., Lackmann C., Barranco A., Ereño Artabe A., Rainieri S., Hollert H., Seiler T.-B. (2019). Pesticides diazinon and diuron increase glutathione levels and affect multixenobiotic resistance activity and biomarker responses in zebrafish (*Danio rerio*) embryos and larvae. Environ. Sci. Eur..

[84] Gutierrez R.M.P., Flores J.M.M., Gonzalez A.M.N. (2018). Anti-inflammatory effect of procumbenoside B from Justicia spicigera on lipopolysaccharide-stimulated RAW 264.7 macrophages and zebrafish model. Pharmacogn. Res..

[85] Oguntibeju O.O. (2019). Type 2 diabetes mellitus, oxidative stress and inflammation examining the links. Int. J. Physiol. Pathophysiol. Pharmacol..

[86] Tingaud-Sequeira A., Ouadah N., Babin P.J. (2011). Zebrafish obesogenic test: A tool for screening molecules that target adiposity. J. Lipid Res..

[87] den Broeder M.J., Moester M.J.B., Kamstra J.H., Cenijn P.H., Davidoiu V., Kamminga L.M., Ariese F., de Boer J.F., Legler J. (2017). Altered Adipogenesis in Zebrafish Larvae Following High Fat Diet and Chemical Exposure Is Visualised by Stimulated Raman Scattering Microscopy. Int. J. Mol. Sci..

[88] Faillaci F., Milosa F., Critelli R.M., Turola E., Schepis F., Villa E. (2018). Obese zebrafish: A small fish for a major human health condition. Anim. Model. Exp. Med..

[89] Oka T., Nishimura Y., Zang L., Hirano M., Shimada Y., Wang Z., Umemoto N., Kuroyanagi J., Nishimura N., Tanaka T. (2010). Diet-induced obesity in zebrafish shares common pathophysiological pathways with mammalian obesity. BMC Physiol..

[90] Meguro S., Hasumura T., Hase T. (2015). Body fat accumulation in zebrafish is induced by a diet rich in fat and reduced by supplementation with green tea extract. PLoS ONE.

[91] Forn-Cuni G., Varela M., Fernandez-Rodriguez C.M., Figueras A., Novoa B. (2015). Liver immune responses to inflammatory stimuli in a diet-induced obesity model of zebrafish. J. Endocrinol..

[92] Turola E., Petta S., Vanni E., Milosa F., Valenti L., Critelli R., Miele L., Maccio L., Calvaruso V., Fracanzani A.L. (2015). Ovarian senescence increases liver fibrosis in humans and zebrafish with steatosis. Dis. Model. Mech..

[93] David C.J., Rajapriya R., Veena V., Kumaresan G. (2016). High Cholesterol Diet Induces Obesity in Zebrafish. Int. J. Adv. Sci. Eng..

[94] Zang L., Shimada Y., Nishimura N. (2017). Development of a Novel Zebrafish Model for Type 2 Diabetes Mellitus. Sci. Rep..

[95] Landgraf K., Schuster S., Meusel A., Garten A., Riemer T., Schleinitz D., Kiess W., Korner A. (2017). Short-term overfeeding of zebrafish with normal or high-fat diet as a model for the development of metabolically healthy versus unhealthy obesity. BMC Physiol..

[96] Clifton J.D., Lucumi E., Myers M.C., Napper A., Hama K., Farber S.A., Smith A.B., Huryn D.M., Diamond S.L., Pack M. (2010). Identification of novel inhibitors of dietary lipid absorption using zebrafish. PLoS ONE.

[97] Zhou J., Xu Y.Q., Guo S.Y., Li C.Q. (2015). Rapid analysis of hypolipidemic drugs in a live zebrafish assay. J. Pharm. Toxicol. Methods.

[98] Schlegel A., Stainer D.Y. (2006). Microsomal triglyceride transfer protein is required for yolk lipid utilization and absorption of dietary lipids in zebrafish larvae. Biochemistry.

[99] Den Dekker A., Davis F.M., Kunkel S.L., Gallagher K.A. (2019). Targeting epigenetic mechanisms in diabetic wound healing. Transl. Res..

[100] Greene D.A., Lattimer S.A., Sima A.A.F. (1987). Sorbitol, phosphoinositides, and sodium-potassium-ATPase in the pathogenesis of diabetic complications. N. Engl. J. Med..

[101] Lee T., Saltsman K.A., Ohashi H., King G.L. (1989). Activation of protein kinase C by elevation of glucose concentration: Proposal for a mechanism in the development of diabetic vascular complications. Proc. Natl. Acad. Sci. USA.

[102] Lioupis C. (2005). Effects of diabetes mellitus on wound healing: An update. J. Wound Care.

[103] Sarras M.P. (2018). Genetic and chemically-induced Zebrafish models for the study of diabetes mellitus. MOJ Anat. Physiol..

[104] Richardson R., Slanchev K., Kraus C., Knyphausen P., Eming S., Hammerschmidt M. (2013). Adult zebrafish as a model system for cutaneous wound-healing research. J. Investig. Derm..

[105] Grada A., Mervis J., Falanga V. (2018). Research Techniques Made Simple: Animal Models of Wound Healing. J. Investig. Derm..

[106] Olsen A.S., Sarras M.P., Intine R.V. (2010). Limb regeneration is impaired in an adult zebrafish model of diabetes mellitus. Wound Repair Regen.

[107] Intine R.V., Olsen A.S., Sarras M.P. (2013). A zebrafish model of diabetes mellitus and metabolic memory. J. Vis. Exp..

[108] Sarras M.P., Leontovich A.A., Olsen A.S., Intine R.V. (2013). Impaired tissue regeneration corresponds with altered expression of developmental genes that persists in the metabolic memory state of diabetic zebrafish. Wound Repair Regen..

[109] Pang S., Gao Y., Wang F., Wang Y., Cao M., Zhang W., Liang Y., Song M., Jiang G. (2020). Toxicity of silver nanoparticles on wound healing: A case study of zebrafish fin regeneration model. Sci. Total Environ..

[110] Dane P.J., Tucker J.B. (1985). Modulation of epidermal cell shaping and extracellular matrix during caudal fin morphogenesis in the zebra fish *Brachydanio rerio*. J. Embryol. Exp. Morph..

[111] Mateus R., Pereira T., Sousa S., de Lima J.E., Pascoal S., Saude L., Jacinto A. (2012). In vivo cell and tissue dynamics underlying zebrafish fin fold regeneration. PLoS ONE.

[112] Niethammer P., Grabher C., Look A.T., Mitchison T.J. (2009). A tissue-scale gradient of hydrogen peroxide mediates rapid wound detection in zebrafish. Nature.

[113] Yoo S.K., Deng Q., Cavnar P.J., Wu Y.I., Hahn K.M., Huttenlocher A. (2010). Differential regulation of protrusion and polarity by PI3K during neutrophil motility in live zebrafish. Dev. Cell.

[114] LeBert D.C., Squirrell J.M., Rindy J., Broadbridge E., Lui Y., Zakrzewska A., Eliceiri K.W., Meijer A.H., Huttenlocher A. (2015). Matrix metalloproteinase 9 modulates collagen matrices and wound repair. Development.

[115] Miskolci V., Squirrell J., Rindy J., Vincent W., Sauer J.D., Gibson A., Eliceiri K.W., Huttenlocher A. (2019). Distinct inflammatory and wound healing responses to complex caudal fin injuries of larval zebrafish. Elife.

[116] Beckman S. (2019). Analyzing Wound Healing in Zebrafish Embryos.

[117] Martin P., Feng Y. (2009). Wound healing in zebrafish. Nature.

[118] Yoo S.K., Lam P.Y., Eichelberg M.R., Zasadil L., Bement W.M., Huttenlocher A. (2012). The role of microtubules in neutrophil polarity and migration in live zebrafish. J. Cell Sci..

[119] Hazlehurst J.M., Woods C., Marjot T., Cobbold J.F., Tomlinson J.W. (2016). Non-alcoholic fatty liver disease and diabetes. Metabolism.

[120] Chen B., Zheng Y.M., Zhang J.P. (2018). Comparative Study of Different Diets-Induced NAFLD Models of Zebrafish. Front. Endocrinol. (Lausanne).

[121] Ahmad O., Wang B., Ma K., Deng Y., Li M., Yang L., Yang Y., Zhao J., Cheng L., Zhou Q. (2019). Lipid Modulating Anti-oxidant Stress Activity of Gastrodin on Nonalcoholic Fatty Liver Disease Larval Zebrafish Model. Int. J. Mol. Sci..

[122] Ma J., Yin H., Li M., Deng Y., Ahmad O., Qin G., He Q., Li J., Gao K., Zhu J. (2019). A Comprehensive Study of High Cholesterol Diet-Induced Larval Zebrafish Model: A Short-Time In Vivo Screening Method for Non-Alcoholic Fatty Liver Disease Drugs. Int. J. Biol. Sci..

[123] Dellambra E., Odorisio T., D’Arcangelo D., Failla C.M., Facchiano A. (2019). Non-animal models in dermatological research. ALTEX.

[124] Aman A.J., Parichy D.M. (2020). Zebrafish Integumentary System. The Zebrafish in Biomedical Research.

